# Epidemiological Characteristics and Trends of Zoonotic Diseases in China from 2015 to 2022

**DOI:** 10.3390/tropicalmed10060159

**Published:** 2025-06-09

**Authors:** Yunfei Zhang, Mengjie Geng, Yue Shi, Baijun Jin, Qian Xiong, Sheng Zhou, Jiangmei Liu, Biao Kan, Maigeng Zhou, Tian Qin, Canjun Zheng

**Affiliations:** 1 National Center for Chronic and Non-Communicable Disease Control and Prevention, Chinese Center for Disease Control and Prevention, Beijing 100050, China; zhangyunfei@icdc.cn (Y.Z.);; 2 National Key Laboratory of Intelligent Tracking and Forecasting for Infectious Diseases, National Institute for Communicable Disease Control and Prevention, Chinese Center for Disease Control and Prevention, Beijing 102206, China; kanbiao@icdc.cn; 3 National Key Laboratory of Intelligent Tracking and Forecasting for Infectious Diseases, Chinese Center for Disease Control and Prevention, Beijing 102206, China; gengmj@chinacdc.cn (M.G.);; 4 Jilin Province for Disease Control and Prevention, Jilin 130062, China; 5 School of Health Policy and Management, Chinese Academy of Medical Sciences and Peking Union Medical College, Beijing 100730, China

**Keywords:** zoonoses, epidemiological characteristics, China

## Abstract

This study aimed to analyze the epidemiological characteristics and incidence trends of zoonotic diseases in China from 2015 to 2022, providing evidence for zoonotic disease prevention and control strategies. Individual case data for nationally reported zoonotic diseases from the Chinese Disease Prevention and Control Information System were collected. Descriptive epidemiology and statistical methods were employed to analyze trends along with changes in their spatial, temporal, and demographic distributions. From 2015 to 2022, the annual incidence rates of leishmaniasis, Japanese encephalitis, rabies, echinococcosis, hemorrhagic fever with renal syndrome (HFRS), and schistosomiasis showed a fluctuating downward trend. The incidence rates of anthrax and leptospirosis remained relatively stable. The incidence of brucellosis declined from 2015 to 2018 but subsequently increased through 2022. Western provinces, such as Gansu, Ningxia, Qinghai, Xinjiang, and Yunnan, remain high-incidence areas for multiple zoonotic diseases, with farmers being the population at the highest risk. These findings indicate it is essential to strengthen cross-sectoral cooperation and develop region- and population-specific prevention and control strategies based on the ‘One Health’ concept, particularly in areas with high incidence rates and among high-risk populations.

## 1. Introduction

Zoonoses refer to diseases and infections that are naturally transmitted between humans and vertebrate animals, caused by common pathogens that are epidemiologically linked [1,2]. These pathogens include bacteria, viruses, parasites, rickettsiae, and others, and are transmitted through various routes such as direct or indirect contact, respiratory, gastrointestinal, and vector-borne pathways. Over 60% of new infectious diseases globally are zoonotic in nature [3,4,5,6]. Zoonoses are widespread worldwide, posing significant threats to public health, economic development, and quality of life [7,8]. According to the World Bank, direct economic losses due to zoonoses exceeded USD 20 billion over the decade, with more than USD 200 billion in indirect losses affecting entire economies [9]. Since 2005, the WHO and partners have convened several meetings to articulate policies for the prevention and control of neglected zoonotic diseases and have included zoonotic diseases, including echinococcosis, schistosomiasis and rabies, in the “Ending the neglect to attain the SDGs: A road map for neglected tropical diseases 2021–2030” [10]. The impact of zoonotic diseases on poor livestock workers in low- and middle-income countries was even more far-reaching, with an additional estimated 2.4 billion cases and 2.7 million deaths per year, and reducing livestock production [2].

As the most populous nation with a vast territory spanning significant latitudinal gradients, China’s demographic and geographic contexts intersect with its status as an agricultural powerhouse. However, the livestock sector—particularly bovine, ovine, and porcine production—remains characterized by limited industrialization, in which smallholder farming persists as a predominant practice [11]. This operational paradigm contributes substantially to the endemic challenges of zoonotic disease transmission, presenting persistent public health concerns. Historically, China has had serious epidemics of schistosomiasis, echinococcosis, leishmaniasis, rabies, leptospirosis, Japanese encephalitis, hemorrhagic fever with renal syndrome (HFRS), and other zoonotic diseases. During the 1950s, about 11.6 million people suffered from schistosomiasis and more than 500,000 cases of leishmaniasis were reported [12,13]. A severe anthrax outbreak in Taihe County, Anhui Province led to over 3000 fatalities [14]. Subsequent public health issues included the 1963 Hebei floods, triggering 140,000 leptospirosis infections, nationwide Japanese encephalitis cases exceeding 170,000 in 1971, and the cases of HFRS surpassing 110,000 in 1986 [15,16]. Since 2018, brucellosis resurgence has been documented, culminating in a historic peak of nearly 70,000 cases by 2022 [17]. These epidemiologically significant zoonoses have been progressively incorporated as notifiable diseases under China’s Law on the Prevention and Treatment of Infectious Diseases.

The occurrence and spread of zoonotic diseases are closely linked to factors such as climate, environmental changes, human–animal contact, and population mobility [18,19,20,21,22]. With the socioeconomic development, transformation of production modes, and elevation of living standards, coupled with marked improvements in ecological and residential environments, the epidemiological characteristics of zoonotic diseases in China have undergone substantial transformations in recent years. This paper analyzes the epidemiological characteristics and trends of zoonotic diseases among notifiable Chinese infectious diseases from 2015 to 2022 based on the China Information System for Disease Control and Prevention. The findings aim to provide evidence for formulating targeted prevention and control strategies.

## 2. Materials and Methods

### 2.1. Data Sources

Individual case data for zoonotic diseases from 2015 to 2022 were obtained from the China Information System for Disease Control and Prevention (CISDCP) Infectious Disease Reporting Information Management System. National population data were sourced from the China Statistical Yearbook published by the National Bureau of Statistics of the People’s Republic of China (https://www.stats.gov.cn/sj/ndsj/ (accessed on 15 October 2024)). Of the 41 infectious diseases legally reportable in China, 12 are zoonotic. These include plague (Class A), hemorrhagic fever with renal syndrome (HFRS), Japanese encephalitis, rabies, anthrax, brucellosis, leptospirosis, highly pathogenic avian influenza in humans, H7N9 avian influenza in humans (Class B), and schistosomiasis, echinococcosis, and leishmaniasis (Class C). This study focuses on nine zoonotic diseases, excluding plague, highly pathogenic avian influenza in humans and H7N9 avian influenza, as these diseases reported fewer than 10 cases annually over the past five years.

### 2.2. Statistical Analysis

The data were organized, categorized, and visualized using Excel 2021 and R software (version 4.3.0). Descriptive and statistical methods were applied to calculate the incidence, incidence rates, proportions, and relative reduction rates for the nine zoonotic diseases from 2015 to 2022. The relative reduction rate was calculated using the following formula [23]:

Relative reduction (%) = 100% × (expected number of cases − observed number of cases)/expected number of cases. Here, the expected number is considered to be the average annual number of cases during 2015 to 2019.

Seasonal indices were employed to analyze seasonal variations in nine zoonotic diseases from 2015 to 2022. The seasonal index for each month was calculated by dividing the monthly average by the overall average.

$$S_{k}=\frac{{\bar{X}}_{k}}{\bar{X}}$$

where

$${\bar{X}}_{k}=\frac{\sum_{i=1}^{n}x_{ik}}{n},\;\bar{X}=\frac{\sum_{i=1}^{n}\sum_{k=1}^{12}x_{ik}}{12\times n}$$

The changing trends in incidence of nine zoonotic diseases from 2015 to 2022 were examined using Joinpoint regression models, implemented through the Joinpoint Regression Software (version 5.0) developed by the Statistical Research and Applications Branch of the National Cancer Institute. This software was utilized to calculate the annual percent change (APC) and average annual percent change (AAPC) [24]. APC is employed to assess the internal trend within each independent interval of the piecewise function or determine a general trend when there are no cut-off points and AAPC is needed to comprehensively evaluate the overall average change trend over multiple intervals [25]. The number, position, and *p*-value of model cut-off points were determined using the Monte Carlo permutation test [26]. If APC or AAPC ≥ 0, it indicates an increasing incidence during that specific time period; if APC or AAPC ≤ 0, it suggests a declining incidence; when APC equals AAPC, it signifies a monotonic general trend without any cut-off points. In addition, it is necessary to test whether both general trends and segmented trends in each period are statistically significant. If *p* < 0.05, it denotes a statistically significant change in the APC or AAPC; if *p* ≥ 0.05, it indicates that the APC or AAPC shows no significant change over the period.

## 3. Results

### 3.1. Incidence Trends

From 2015 to 2022, the annual incidence rates of leishmaniasis, Japanese encephalitis, rabies, echinococcosis, HFRS and schistosomiasis all exhibited a declining trend, and the AAPC was −11.48 (*p* < 0.001), −27.45 (*p* = 0.005), −6.36 (*p* < 0.001), −6.04 (*p* = 0.390) and −63.93 (*p* = 0.155), respectively. The annual incidence rates of anthrax and leptospirosis remained relatively stable, consistently fluctuating between 0.02 and 0.03 per 100,000, and 0.01 and 0.03 per 100,000, respectively. The annual incidence rate of brucellosis decreased from 4.18 per 100,000 in 2015 to 2.73 per 100,000 in 2018 before increasing to 4.69 per 100,000 in 2022, with APCs of −13.45 (*p* = 0.048) from 2015 to 2018 and 17.89 (*p* = 0.022) from 2018 to 2022.

**Figure 1:**
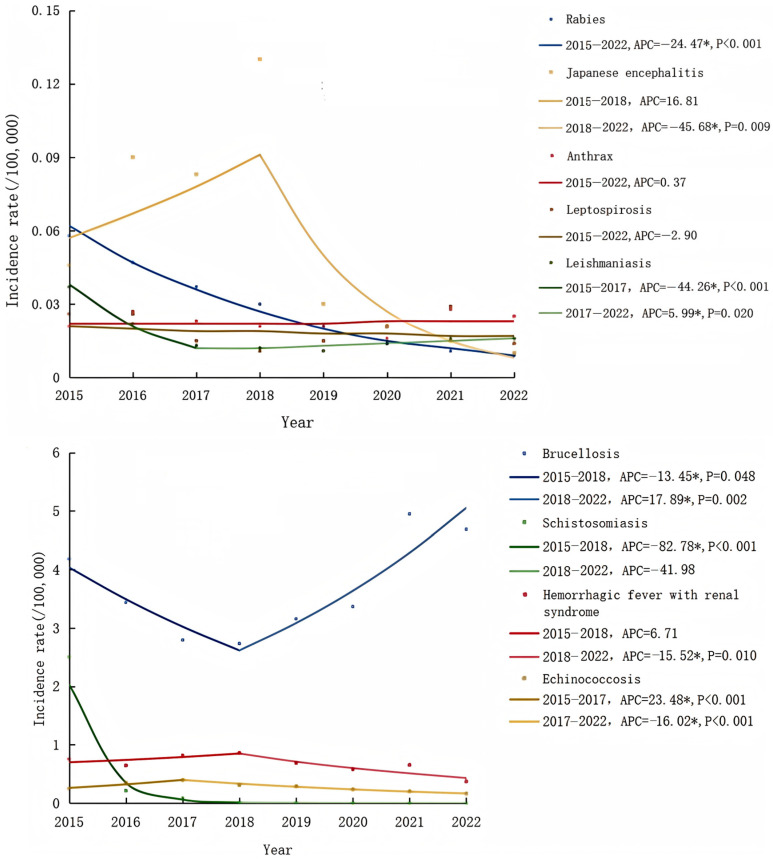
Joinpoint regression curves for the incidence rates of nine zoonotic diseases from 2015 to 2022. (*) Indicates statistically significant results (*p* < 0.05).

For detailed trends and statistical values, please refer to Figure 1.

Among the nine zoonotic diseases, brucellosis and leptospirosis exhibited significant increases in their average annual cases, with reduction rates of −35.87% and −15.79%, respectively, from 2020 to 2022 compared to the period from 2015 to 2019. In contrast, the number of cases of the other seven zoonotic diseases declined during the same period. Notably, brucellosis showed year-on-year increases of 5.15%, 55.27%, and 47.19% in 2020, 2021, and 2022, respectively, relative to its average annual cases from 2015 to 2019. For detailed data, refer to Table 1.

### 3.2. Population Distribution

The age distribution of reported cases from 2015 to 2022 reveals that the highest proportion of cases for schistosomiasis, echinococcosis, hemorrhagic fever, brucellosis, leptospirosis, and anthrax occurs in the 40–59 age group, with percentages of 61.17%, 40.84%, 45.73%, 52.74%, 38.09%, and 46.59%, respectively.

Both Japanese encephalitis and leishmaniasis exhibit two high-incidence age groups. Japanese encephalitis predominantly affects children under 10 years old and individuals aged 60–79, accounting for 27.47% and 23.38%, respectively. Leishmaniasis primarily impacts children under 4 years old and individuals aged 40–59, making up 43.97% and 22.58% of cases, respectively. Rabies is most prevalent in individuals aged 40–59 and 60–79, which represent 34.63% and 39.15% of cases, respectively.

**Figure 2:**
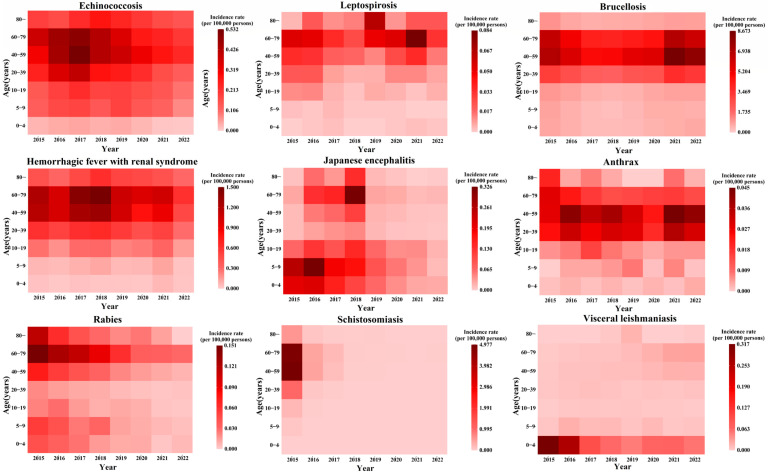
Age distribution of nine zoonotic diseases from 2015 to 2022.

**Figure 3:**
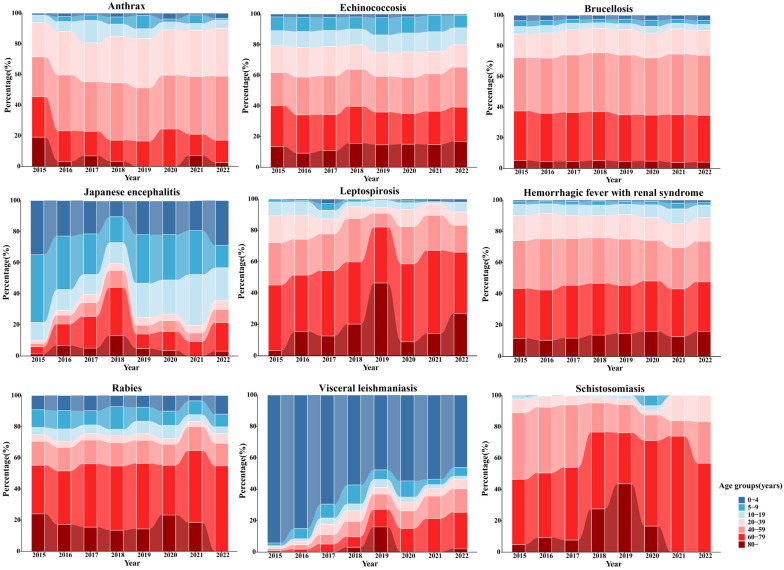
Proportional distribution of nine zoonotic diseases by age group from 2015 to 2022.

A trend analysis of the age distribution of reported cases from 2015 to 2022 indicates a significant increase in the proportion of cases in older age groups for zoonotic diseases such as Japanese encephalitis, hemorrhagic fever, brucellosis, leptospirosis, anthrax, rabies, and schistosomiasis (χ^2^ test, *p* < 0.05). Detailed distributions are presented in Figure 2 and Figure 3.

### 3.3. Occupational Distribution

The occupational distribution of reported cases from 2015 to 2022 indicates that farmers represent more than half of the cases of schistosomiasis, brucellosis, leptospirosis, rabies and hemorrhagic fever with renal syndrome, with proportions of 93.62%, 80.21%, 75.19%, 74.33% and 68.90%, respectively, making them the most affected occupation in these diseases.

For anthrax and echinococcosis, both farmers and herders show relatively high proportions of cases. Farmers account for 38.59% and 45.20% of cases, respectively, while herders represent 51.88% and 25.64%. In the case of leishmaniasis, farmers and scattered children (children not enrolled in kindergarten but receiving home care) contribute 30.04% and 49.17% of the cases, respectively. For Japanese encephalitis, farmers and students make up 44.03% and 23.71% of cases.

**Figure 4:**
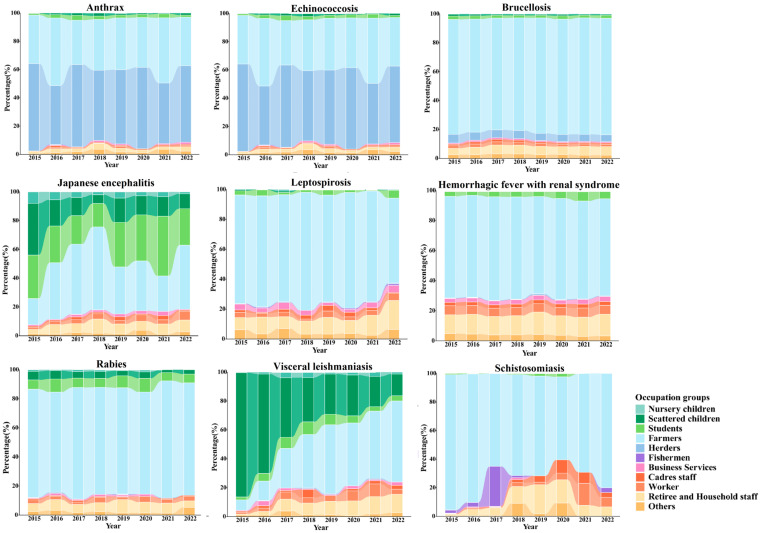
Trends in occupational distributions for nine zoonotic diseases from 2015 to 2022.

While the occupational distribution of most zoonotic diseases has remained relatively stable, notable changes have occurred in the distribution of Japanese encephalitis and leishmaniasis cases. The proportion of students, scattered children, and preschool children in Japanese encephalitis cases has gradually decreased from 30.29%, 35.90%, and 8.01% in 2015 to 25.34%, 10.27%, and 1.37% in 2022, respectively. Similarly, the proportion of scattered children in leishmaniasis cases has decreased dramatically from 86.51% in 2015 to 15.38% in 2022. See Figure 4.

### 3.4. Seasonal Distribution

**Figure 5:**
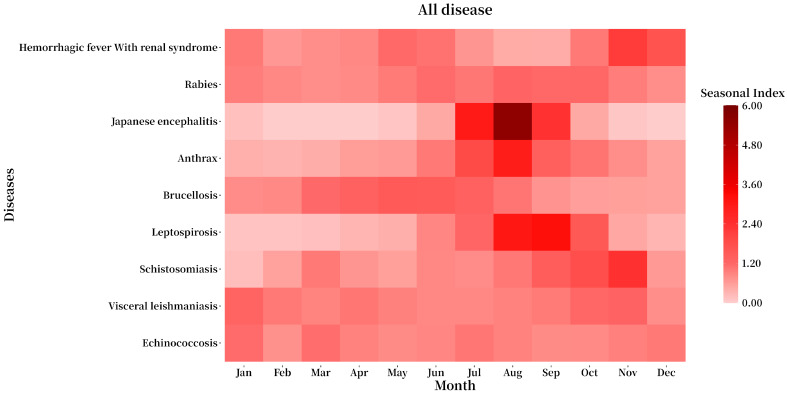
Seasonal index changes for prevalence of nine zoonotic diseases from 2015 to 2022.

**Figure 6:**
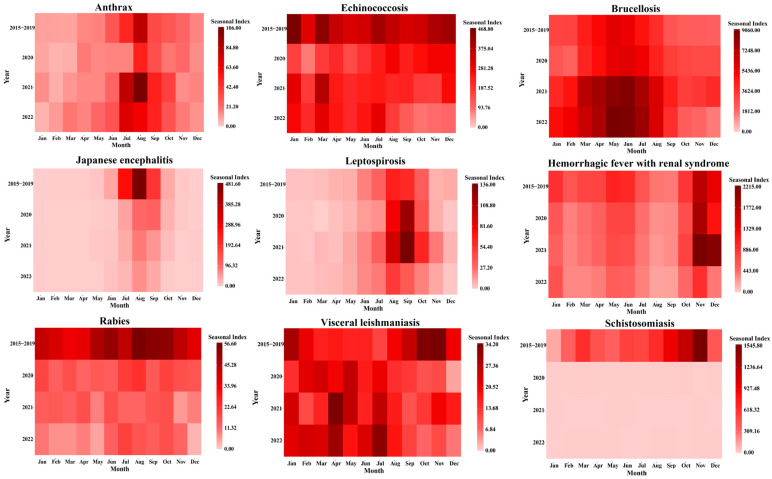
Seasonal indexes of the prevalence of nine zoonotic diseases from 2020 to 2022 compared to the average from 2015 to 2019.

From 2015 to 2022, except for echinococcosis, which showed no clear seasonality, the other eight zoonotic diseases exhibited significant seasonal variation in incidence. Specifically, brucellosis (from March to August), rabies (from June to October), Japanese encephalitis (from July to September), anthrax (from July to October), leptospirosis (from July to October), and schistosomiasis (from September to November) all demonstrated a single seasonal peak. HFRS, on the other hand, had two distinct peaks: one in spring–summer (from April to June) and another in autumn–winter (from October to January of the following year). See Figure 5 and Figure 6.

### 3.5. Regional Distribution

From 2015 to 2022, zoonotic diseases exhibited distinct regional patterns, with certain provinces showing notably higher incidences.

The provinces with a high incidence of HFRS were primarily Shaanxi, Heilongjiang, Liaoning, Jilin, and Jiangxi. Notably, the incidence in Shaanxi fluctuated between 2.46 and 7.63 per 100,000 during the study period.

The provinces with high incidences of Japanese encephalitis included Gansu, Ningxia, Shaanxi, Yunnan, and Shanxi, with annual incidence rates of 0.51 per 100,000, 0.32 per 100,000, 0.30 per 100,000, 0.14 per 100,000, and 0.09 per 100,000, respectively.

Provinces with a high incidence of anthrax included Qinghai, Tibet, Gansu, Ningxia, and Inner Mongolia, with annual incidence rates of 1.18 per 100,000, 0.41 per 100,000, 0.27 per 100,000, 0.23 per 100,000, and 0.09 per 100,000, respectively.

Brucellosis was predominantly concentrated in Inner Mongolia, Ningxia, Xinjiang, Shanxi, and Heilongjiang. In Inner Mongolia and Ningxia, the incidence rate showed a significant increase, rising from 28.90 per 100,000 and 43.66 per 100,000 in 2015 to 75.16 per 100,000 and 84.80 per 100,000 in 2022.

For echinococcosis, the provinces with high-incidence provinces were Qinghai, Xinjiang, Tibet, Ningxia, and Gansu, with annual incidence rates of 15.70 per 100,000, 6.06 per 100,000, 4.61 per 100,000, 3.05 per 100,000, and 1.12 per 100,000, respectively.

Leishmaniasis had a high incidence in Xinjiang, Gansu, Shanxi, Shaanxi, and Sichuan, with annual incidence rates of 0.36 per 100,000, 0.17 per 100,000, 0.15 per 100,000, 0.06 per 100,000, and 0.16 per 100,000, respectively.

Leptospirosis showed a higher incidence in central and southern China, particularly in Fujian, Yunnan, Zhejiang, Hunan, and Sichuan, with annual incidence rates of 0.07 per 100,000, 0.07 per 100,000, 0.06 per 100,000, 0.05 per 100,000, and 0.05 per 100,000, respectively.

Schistosomiasis had higher incidence rates in Anhui, Hunan, Jiangxi, Hubei, and Yunnan, with annual incidence rates of 5.01 per 100,000, 1.31 per 100,000, 1.27 per 100,000, 0.49 per 100,000, and 0.82 per 100,000, respectively.

Rabies was most prevalent in central and southern China, particularly in Hunan, Guizhou, Guangxi, Yunnan, and Henan. Notably, the incidence rate of rabies declined consistently each year from 2015 to 2022.

**Figure 7:**
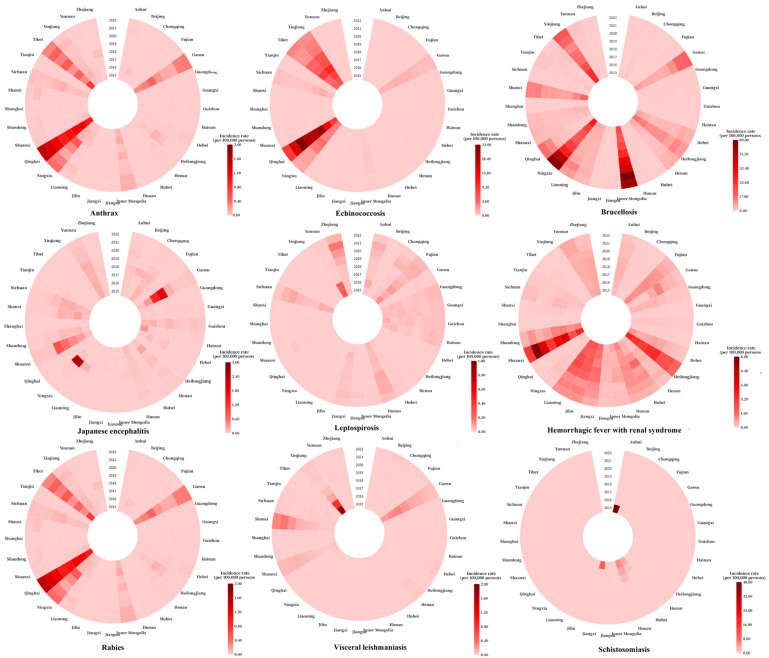
Incidence rate of nine zoonotic diseases in provinces in China from 2015 to 2022.

See Figure 7 for a detailed overview.

## 4. Discussion

Zoonoses are infectious diseases that can be transmitted between animals and humans. The incidence of zoonotic diseases in humans is often influenced by similar trends in animal populations. These diseases are also closely linked to factors such as climate, agricultural practices, trade in livestock and animal products, human lifestyle, hygiene awareness, and public health interventions. Zoonotic diseases vary in terms of their pathogens, transmission routes, host species, and geographic distribution, leading to significant regional and demographic differences. This paper examines the distribution patterns, and trends between 2015 and 2022 on nine zoonotic diseases in China.

In recent years, zoonotic diseases such as schistosomiasis, rabies, echinococcosis, leishmaniasis, Japanese encephalitis, and hemorrhagic fever with renal syndrome have shown fluctuating downward trends. These trends can largely be attributed to sustained socio-economic development, increased government investment in public health, and enhanced preventive measures, including improved monitoring and vaccination programs. For instance, schistosomiasis, through decades of efforts such as chemotherapy for humans and animals, snail control, and health education, was brought under control nationwide by 2015. By 2020, the goal of schistosomiasis elimination was achieved, and reported cases decreased to minimal levels. Since then, only sporadic cases have been recorded in a few southern provinces [22,27]. Echinococcosis, primarily prevalent in pastoral areas of western China (e.g., Qinghai, Tibet, Sichuan, Xinjiang, Gansu, and Ningxia), has also seen a gradual decrease in incidence since 2015. This decrease can be attributed to targeted prevention strategies, such as dog deworming, population screening, and livestock immunization [28]. Rabies cases in China have steadily declined since 2006, from a peak of 3308 cases to just 126 cases in 2022. This decline has been facilitated by strengthened dog management, the promotion of post-exposure prophylaxis, and widespread vaccination campaigns. Rabies cases are now primarily concentrated in Henan, Hubei, and Hunan provinces, with most regions reporting only sporadic incidents [29,30]. Since the Japanese encephalitis vaccine was included in the national immunization program in 2008, the incidence of Japanese encephalitis has decreased significantly in China. However, in recent years, there has been an increasing proportion of elderly cases in central and western provinces. With improved living conditions and reduced exposure to host animals, along with increased vaccination coverage [31,32], HFRS has shown a generally declining trend since the 1980s. Most provinces maintain low-level fluctuations, while Shaanxi and northeastern regions have seen relatively higher incidences [33,34]. Anthrax, which was once a significant public health concern in China, has seen a steady decline since the 1990s. From 2015 to 2022, the annual number of cases fluctuated below 500, with most cases occurring in the western and northeastern provinces. However, there has been an increase in reported cases in central provinces such as Henan, Shanxi, and Shandong [35]. Leptospirosis has remained at low levels overall, with high-incidence areas remaining relatively stable in provinces along the Yangtze River [36]. The incidence of leishmaniasis continued to decline in Xinjiang, Gansu, Sichuan and other traditional epidemic areas, but rebounded in Shanxi, Shaanxi, Henan, Hebei and other provinces that had not reported cases for many years. Meanwhile, the incidence of canine visceral leishmaniasis increased year by year, with the epidemic continued to spread [37,38]. In recent years, due to the increasing number of livestock breeding such as cattle and sheep in China, the high proportion of rural free-range farming, the frequent and expanded range of long-distance livestock trading, and the general lack of awareness of infectious disease protection among residents, the epidemic of brucellosis in China has risen significantly, with the incidence rising from 2.73/100,000 in 2018 to 4.69/100,000 in 2022. The incidence of brucellosis has continued to increase in the previously high prevalence areas and has spread to the southern provinces [39,40].

Zoonoses are usually characterized by an endemic distribution pattern. Influenced by livestock breeding, vector distribution and other factors, the incidence rates of different Zoonoses vary widely across China. Among them, brucellosis is mainly prevalent in northern provinces such as Inner Mongolia, Xinjiang, Ningxia, and Gansu, mainly because these regions are important sheep breeding areas in China, and sheep are the main source of brucellosis infection in China. The high incidence of echinococcosis in the pastoral areas of the Qinghai–Tibet Plateau is mainly related to the large numbers of yaks and dogs raised by local herdsmen. Yaks and dogs form a transmission cycle of echinococcosis in this area. Anthrax is also mainly prevalent in pastoral areas of the Qinghai–Tibet Plateau, with yaks being the main source of infection in the area. Leishmaniasis is prevalent in provinces such as Xinjiang, Gansu, and Sichuan, mainly due to the widespread distribution of sand flies. In addition, schistosomiasis is mainly prevalent in the middle and lower reaches of the Yangtze River in southern China, which is related to the suitability of the intermediate host of schistosomiasis—snails— for breeding in this area. However, due to strong control measures, schistosomiasis is approaching the elimination target in China. This study identified Inner Mongolia, Xinjiang, Gansu, Ningxia, and Sichuan provinces in the northwest and northern regions as high-risk areas for zoonotic diseases such as brucellosis, anthrax, echinococcosis, and leishmaniasis.

Farmers, particularly those engaged in livestock farming and outdoor agricultural activities, constitute a high-risk group for zoonotic diseases due to their frequent contact with animals. The most affected age groups are middle-aged and elderly individuals, as these populations are predominantly engaged in rural farming activities. In recent years, there has been an increasing trend of zoonotic disease cases among individuals aged 60 and above. This may be due to younger populations migrating to cities for work, leaving older individuals as the primary labor force in rural farming. This age group, often lacking awareness and preventive measures, is particularly vulnerable to zoonotic diseases.

As livestock farming continues to develop and trade in livestock products increases, the geographic spread of zoonoses is expanding. For example, anthrax outbreaks are gradually spreading from the western provinces to neighboring central and eastern regions including Hebei, Shanxi, Henan, and Shandong, while brucellosis is also expanding further south. Echinococcosis remains primarily confined to western China, where its complex transmission cycle involves definitive hosts such as dogs, wolves, and foxes. Leishmaniasis, once largely confined to the western provinces, is now spreading toward central regions like Henan, Shaanxi, Shanxi, and Hebei, as sandfly populations and host animals expand their ranges.

## 5. Conclusions

The western and northern regions of China, including Inner Mongolia, Xinjiang, Gansu, Ningxia, and Qinghai, remain high-risk areas for zoonotic diseases, with residents facing threats from multiple zoonotic infections. The rise in livestock farming, increased trade in animal products, and changes in environmental conditions have contributed to the geographic spread of diseases such as brucellosis, anthrax, and leishmaniasis.

The control of zoonoses requires close cooperation between human and animal health authorities and other relevant authorities for disease prevention and control to effectively reduce the risk of zoonoses occurrence and transmission [7,41,42].

It is essential to implement multiple measures according to the “One Health” approach [4,43,44]. With reference to the One Health Action Plan proposed by WHO [45], we should enhance multi-sectoral collaboration and cross-regional coordination. The top priority is for health, animal husbandry, forestry and other departments to jointly carry out surveillance of humans, livestock and wild animals, share monitoring information, and promptly detect and control animal and human infections. At the same time, for livestock such as cattle and sheep that can serve as hosts for various zoonotic diseases, breeding enterprises or individuals must strengthen scientific breeding and management practices. In addition, the health department and animal husbandry authorities need to strengthen the vaccination programs for diseases such as rabies, Japanese encephalitis, hemorrhagic fever with renal syndrome, leptospirosis, brucellosis, and anthrax, and improve the vaccination coverage for both animals and human populations. The health department strengthens health education for high-risk populations and enhances their personal protective awareness; In response to the spread of zoonotic diseases such as brucellosis, animal husbandry departments must strengthen supervision and quarantine of animal trade to reduce cross-regional transmission. By implementing these strategies, China can further reduce the incidence of zoonoses and curb its spread under a “one health” framework.

## Figures and Tables

**Table 1:** Comparison of the incidence number of nine zoonotic diseases during 2020–2022 with the average annual number during 2015–2019 in China.

Diseases	Relative Reduction (%) (2015–2019 vs. 2020)	Relative Reduction (%) (2015–2019 vs. 2021)	Relative Reduction (%) (2015–2019 vs. 2022)	Relative Reduction (%) (2015–2019 vs. 2020–2022)
Rabies	62.21	70.63	75.12	69.32
Anthrax	30.56	−21.51	−8.18	0.29
Japanese encephalitis	72.43	80.19	86.03	79.55
Brucellosis	−5.15	−55.27	−47.19	−35.87
Hemorrhagic fever with renal syndrome	21.90	11.65	49.82	27.79
Leptospirosis	−15.93	−57.30	25.84	−15.79
Leishmaniasis	22.61	11.88	13.41	15.96
Schistosomiasis	99.44	99.83	99.61	99.63
Echinococcosis	24.42	36.42	46.82	35.89

## Data Availability

Data supporting this study’s findings are accessible from the corresponding authors on reasonable request.

## Abbreviations

The following abbreviations are used in this manuscript:

HFRS	Hemorrhagic fever with renal syndrome
CISDCP	China Information System for Disease Control and Prevention
APC	Annual percent change
AAPC	Average annual percent change
